# Subcutaneous Semaglutide during Breastfeeding: Infant Safety Regarding Drug Transfer into Human Milk

**DOI:** 10.3390/nu16172886

**Published:** 2024-08-28

**Authors:** Hanin Diab, Taylor Fuquay, Palika Datta, Ulrich Bickel, Jonathan Thompson, Kaytlin Krutsch

**Affiliations:** 1 School of Veterinary Medicine, Texas Tech University, Amarillo, TX 79106, USAjon.thompson@ttu.edu (J.T.); 2 School of Medicine, Texas Tech University Health Sciences Center, Amarillo, TX 79106, USApalika.d.datta@ttuhsc.edu (P.D.); 3 Jerry H. Hodge School of Pharmacy, Texas Tech University Health Sciences Center, Amarillo, TX 79106, USA; ulrich.bickel@ttuhsc.edu

**Keywords:** ozempic, semaglutide, wegovy, rybelsus, lactation, breastfeeding, pharmacology, GLP-1 receptor, GLP-1 analogue, infant risk

## Abstract

Postpartum mothers and their healthcare providers often face the challenge of limited data regarding the safety of drug therapies during lactation. Pregnancy can lead to sustained weight gain, and obesity can negatively impact both physical and psychological well-being. The introduction of GLP-1 agonists to augment weight loss has become a topic of interest for many postpartum mothers. Our study aims to investigate the transmission of semaglutide into human milk in the first steps to ensure the safety and health of both lactating mothers and their breastfed infants. Semaglutide quantification was performed using high-resolution liquid chromatography-mass spectrometry. InfantRisk Center Human Milk biorepository released milk samples from eight women collected at 0, 12 and 24 h post-semaglutide administration. Semaglutide was extracted using protein precipitation in methanol, followed by chromatographic separation. Linear calibration curves for the method ranged between 2.5–30 ng/mL, with a limit of detection of 1.7 ng/mL and a limit of quantification of 5.7 ng/mL (LLOQ). Semaglutide was not detected in any of the collected human milk samples. A worst-case scenario of the relative infant dose (RID) was calculated using the LLOQ as the drug concentration in milk when considering semaglutide’s bioavailability and long-acting dose profile. The maximum RID projected was 1.26%, far below the standard 10% safety threshold. While questions about long-term infant outcomes, the safety of maternal nutrient intake, and the nutrient content of breast milk remain, our findings suggest that semaglutide concentrations in human milk are unlikely to pose clinical concerns for breastfed infants. These results support healthcare providers in making informed decisions regarding postpartum therapeutic interventions.

## 1. Introduction

Breast milk is universally regarded as the best source of nutrition for infants [1]. Research has demonstrated that human milk contains various bioactive compounds that influence gastrointestinal and immune system function, as well as brain development [2]. Further, breast milk is believed to exhibit dynamic changes in composition to support infant health, and such changes are impossible to simulate with infant formula [3]. Remarkably, recent studies have suggested that breastfeeding reduces the maternal risk of developing metabolic disorders later in life, particularly protecting against obesity and type 2 diabetes in adulthood [4,5]. Besides its nutritional and physical benefits, breastfeeding is cost-effective, convenient, and fosters bonding between mother and child.

Pregnancy often involves a significant weight increase. Maternal weight gain surpassing the recommendations set by the Institute of Medicine (I.O.M., U.S.A.) is a significant factor contributing to persistent obesity among women [6,7]. Women, after pregnancy, usually show a weight change. For example, Chu et al. found that around 40% of women with a normal weight before pregnancy and 60% of women classified as overweight experienced excessive weight gain during their pregnancy [8]. Studies with a longer follow-up period of fifteen years after delivery showed that compared with 60% of normal-weight women, only 35% of overweight women returned to within 1.5 kg of their pre-pregnancy weight. Furthermore, studies have shown that retaining 3 or more body mass index units during a 2-year period between births has a greater risk of adverse pregnancy outcomes for both the mother and infant [9].

Semaglutide acts as an incretin mimetic, which binds glucagon-like peptide-1 (GLP-1) receptors, serving as an anti-obesity medication in addition to a therapeutic option for Type II diabetes. The natural GLP-1 hormone consists of approximately 30 amino acids which acts to inhibit glucagon release from the alpha cells of the islets of Langerhans in the pancreas. Semaglutide has a 94% structural similarity to human GLP-1, its primary difference being an elongated hydrophilic spacer to extend the molecule’s lifespan via albumin binding. The drug has a binding affinity of 0.38 ± 0.06 nM for the GLP-1 receptor [10]. Semaglutide was formulated with a C-18 fatty di-acid chain, promoting albumin binding and extending the half-life of the product compared with the GLP-1 hormone, allowing for a weekly dosing schedule. Semaglutide is an injectable product due to its limited oral bioavailability, yet, infant exposure to semaglutide in milk is via the oral route.

Interestingly, human GLP-1 is present in breastmilk, and some authors have postulated it serves to satiate an infant since measured levels were higher in hindmilk compared to foremilk [11,12]. The mechanism by which GLP-1 is present in human milk has yet to be explored. A potential complication of breastfeeding occurs when pharmaceuticals consumed by the mother can transfer into the milk supply and be indirectly transmitted to the infant during feeding. Young infants have different metabolic capacities than adults, and limited exposure to hazardous substances can result in infant harm. This is especially relevant in the case of semaglutide, given its structural similarity to the natural GLP-1 hormone present in milk, compounded by the potential for early infant satiety with drug exposure. Given semaglutide’s structural similarity to GLP-1, the presence of GLP-1 analogs in milk via active transport is a possible infant health concern.

The focus of the current manuscript is to assess the transfer of semaglutide into the maternal milk supply post-subcutaneous administration of the drug. The drug formulation, route, and dose are highly relevant to this discussion, regardless of the indicated use of the drug by the mother.

All formulations require a dose titration due to gastrointestinal side effects. Semaglutide, developed by Novo Nordisk under the brand name Ozempic for type 2 diabetes, is administered as a once-weekly subcutaneous injection with a starting dose of 0.25 mg to a maximum dose of 2 mg per week. The semaglutide peptide is poorly absorbed following oral administration due to acidic degradation and proteolytic enzymes in the stomach [13]. Rybelsus is an oral tablet form of semaglutide intended for type 2 diabetes. Semaglutide tablets are formulated with a permeation enhancer, SNAC, to overcome the low oral bioavailability of semaglutide due to protein degradation in the gastrointestinal tract [14]. Even with these modifications, the daily dose of semaglutide tablets is 3 mg daily (starting titration) to a maximum of 14 mg per day, almost a 500% increase in active drug taken per week at the maximum dose [15]. As such, even when formulated with a gastric epithelial permeability enhancer (SNAC), oral doses of semaglutide totaled over a week are 36 to 49 times higher than the weekly injectable doses of semaglutide to overcome the bioavailability limitation [16]. Even then, mean serum doses of oral semaglutide are considerably lower than the subcutaneously administered drug in adult populations [17]. WEGOVY is another noteworthy product in the semaglutide family. It is the identical molecule in Ozempic but is indicated for appetite reduction and weight loss at a higher maximum dose of 2.4 mg subcutaneously per week [18]. Given its implications for treating diabetes and obesity, semaglutide has become a remarkably popular drug, with current sales revenues of over 12 billion USD and projected to increase by >20% each year through 2026 [19].

Indeed, lactating mothers have expressed overwhelming interest in taking semaglutide while breastfeeding to control weight [20]. Drug information calls pertaining to semaglutide use during lactation to the InfantRisk Call Center at Texas Tech University HSC increased by over 500% from 2021 to 2024 (K. Krutsch, InfantRisk Center Data, 24 June 2024). Given the emergence of semaglutide interest in this population, studying its presence in human milk and its impact on infant nutrition is a vital and emerging area of research.

The effects of semaglutide use on human milk are potentially far-ranging and complex. Semaglutide’s predominant impact on weight loss is achieved by decreasing energy intake with limited influence on energy expenditure [21]. Notably, semaglutide goes beyond caloric reduction. It also reduces hunger, food cravings, and heightened sensations of fullness, ultimately improving eating control. Studies on rodents indicate that semaglutide affects neural pathways governing appetite regulation [22]. Moreover, the slowed gastric emptying process induced by semaglutide can lead to early satiety and reduced appetite, consequently decreasing food intake and positively impacting blood glucose levels and weight loss [23]. While these outcomes may be perceived as positive for weight loss, they may be incompatible for an infant exposed to the drug via milk as well as incompatible with the increased maternal nutrient demand that accompanies lactation. Exclusive breastfeeding is more nutritionally demanding than late pregnancy (up to an additional 500 kcal per day), as the lactating mother continues to provide the entirety of the infant’s nutrient needs for the first six months of life. Therefore, the use of semaglutide during lactation may pose substantial complications regarding infant well-being, which must be managed.

This work solely considers the quantity of semaglutide transferred into breast milk from a limited number of healthy mothers undergoing self-administered, sub-cutaneous application of the drug. The study included a cohort of 8 women; samples were collected 0, 12, and 24 h post-administration of semaglutide. A method employing liquid chromatography (LC) coupled to high-resolution mass spectrometry (MS) was employed for the measurement of semaglutide in human milk samples. Relative infant doses (RID) were computed to assess infant risk solely due to the molecular transfer of the drug into breast milk.

## 2. Materials and Methods

### 2.1. Sample Acquisition, Dosing, and Reportable Info about Participating Women

This study utilized data collected from the InfantRisk Human Milk Biorepository (HMB) which has been approved on 22 February 2021 by the institutional ethics committee (A21-4214). The biorepository targets the collection of milk samples and self-related health information for mother and infant dyad via a questionnaire. Achieving steady state conditions with semaglutide, following standard dose titration schedules, can take more than four months after drug initiation. The weekly administration, inconsistent titration schedules, and the risk of breastfeeding cessation during this extended period posed significant challenges in collecting milk samples at steady state. Balancing these risks while ensuring the collection of informative samples was crucial. Therefore, we prioritized collecting samples immediately before any dose changes, even if this occurred prior to reaching steady state concentrations, to maximize the informativeness of our analysis. Participants were asked to express milk samples after a minimum of three administrations of the same dose. Milk samples collected from 0, 12, and 24 h post-administration and their corresponding de-identified health questionnaires were released for 8 different individuals from the HMB for this study. If milk samples for multiple doses were provided, samples were only released from the biorepository for the highest dose collected closest to steady-state.

### 2.2. Sample Preparation

An aliquot of milk was obtained, and a 0.5 mL sample dispensed into a 2 mL microcentrifuge vial containing 0.5 mL of chloroform (ACS grade, LabChem, Zelienople, PA, USA). The vial was vortexed and centrifuged at 2000× *g* for 30 s at room temperature to separate the aqueous and organic layers. Then, 0.4 mL of the the upper (milk) fraction was pipetted and mixed with 1.2 mL of methanol (LC-MS grade, Fisher, Fair Lawn, NJ, USA). This process promotes protein precipitation. The vials were centrifuged at 2000× *g* for 5 min at room temperature, and 1.5 mL of supernatant was collected. Partial evaporation continued until only ~1 mL of fluid remained in the vial and finally water was added to get the final volume to 2 mL for LC-MS analysis.

External calibration standards were prepared within breast milk matrix by spiking 0.5 mL of milk with varying volumes (1, 3, 5, 8, 12 μL) of 1250 ng/mL semaglutide stock solution dissolved in methanol. The breast milk was known to be semaglutide free. This results in semaglutide standard concentrations of 2.5, 7.5, 12.5, 20, and 30 ng/mL within the milk matrix. Then, these standards were treated according to the sample preparation protocol outlined in the preceding paragraph prior to analysis via LC-MS. The semaglutide standard added to human milk as a calibrant ex vivo is assumed to behave exactly like semaglutide present in human milk of mothers taking the drug. There may be inaccuracies in calibration and measurement if the calibration standards do not interact with the milk matrix’s fat and protein components in the same way as in vivo occurring semaglutide does. The reliability of our quantitative results would be considerably impacted if such differences exist.

### 2.3. Liquid Chromatography (LC)/Mass Spectrometry (MS)

Chromatography was accomplished through use of an Ultimate 3000 chromatograph (ThermoFisher Scientific, Waltham, MA, USA) equipped with a binary nanoflow pump. Mobile phase solvent A was LC/MS grade water with 0.1% formic acid added. Mobile phase solvent B was LC/MS grade acetonitrile with 0.1% formic acid added. A constant flow rate of 0.6 μL/minute was used throughout experiments. Gradient elution was employed beginning with 5% B during the initial 2 min of the separation. Then, the mobile phase was linearly ramped to 16% B from 2–3.5 min. A separate linear ramp from 3.5–9.9 min transitioned to 95% B (acetonitrile). The gradient was held at 95%B until the 15-min mark when it was restored to 5% B until the 20-min run time elapsed. The final 5 min at 5%B was meant to recondition the column and prepare for next run. The autosampler needle was washed between samples with 60 μL of 75% methanol with 25% water. The chromatography column was a 75-micron i.d. x 150 mm length Magic3 C-18 column packed with 1.8 micron particles with pore size of 100 Angstrom (PremierLCMS, P/N FN2-25113). Column eluant was subjected to electrospray ionization through a liquid-junction source (Nanoflex, Thermo Scientific) using a pulled fused silica capillary emitter (Lotus, Fossil IonTech, Madrid, Spain) at ESI voltage of 2.3 kV.

Mass spectrometery was performed on a Q-Exactive HF Orbitrap mass spectrometer (ThermoFisher Scientific). Positive ion mode spectra were collected using parallel reaction monitoring (PRM) mode [24,25]. In this approach, the inlet quadrupole of the mass spectrometer is used to isolate selected analyte ions followed by collision induced dissociation and collection of MS/MS spectra. The method can be multiplexed to select more than one precursor, and this option was employed for analysis (*n* = 2 precursors). The MS method selected semaglutide parent ions at *m*/*z* = 1029.286 (z = +4) and *m*/*z* = 1372.047 (z = +3) for fragmentation with a quadrupole isolation window of 2.0 Da *m*/*z* and offset of 0.4 Da *m*/*z*. A normalized collision energy of 35 was used for CID fragmentation, prior to MS2 occurring with Rs = 30,000, AGC target of 5E6, and maximal integration time of 140 ms selected. The fragment ion noted at *m*/*z* = 960.410 + 5 m.m.u. (z = +1) was used for quantitation through noting peak area using commercial software (Freestyle v.1.7, ThermoFisher Scientific).

### 2.4. Estimation of Relative Infant Dose (%)

The maternal weight-adjusted infant dose, the relative infant dose (RID%), is often used to assess infant exposure to drugs through breastfeeding. This parameter is the percentage of an infant dose of drug via a full diet of human milk relative to the maternal dose (mg/kg) over the same period. An RID of >0% is often considered a safety threshold in risk assessments [26,27,28].

(1) $$RID\;(\%)=100\;\times \;\frac{infant\;daily\;oral\;dose\;per\;kg\;from\;milk}{maternal\;daily\;dose\;per\;kg}$$

The fraction’s numerator is computed as the product of the measured drug concentration present in milk times estimated milk intake of 150 mL/kg/day [29,30].

The RID calculation was adjusted to include parameters for bioavailability and extended dosing intervals, which will improve the interpretation of RID in the setting of subcutaneous semaglutide injection.

First, we will contextualize bioavailability in the RID by including these parameters in the RID calculation (RID_F_). The subcutaneous bioavailability of semaglutide is reported as 89% [31]. The oral bioavailability of semaglutide is low but unknown per a medical information request at Novo Nordisk (B. Shah, personal communication, 20 June 2024). As such, oral bioavailability will be overestimated using the available parameter for semaglutide formulated with the permeability enhancer, SNAC (F < 0.01), though SNAC will not be present in this scenario [32]. This overestimate will assist in contextualizing a “worst-case scenario” of infant systemic exposure to semaglutide via milk in the setting of maternal subcutaneous use.

Next, we will add an adjustment for the long-acting formulation. Though RID calculations for long-acting injectables sometimes use the entirety of the maternal dose in the denominator compared to a day’s infant milk consumption, we believe this will inappropriately underestimate the relative dose per day. To combat this underestimate, we will convert the weekly dose to an equivalent daily dose.

(2) $${RID}_{F}(\%)=100\;\times \;\frac{infant\;daily\;oral\;dose\;per\;kg\;from\;milk\;\times \;F(oral)}{\;\frac{1}{7}\;maternal\;weekly\;subcutaneous\;dose\;per\;kg\;\times \;F(S.C.)}$$

The assumptions remain the same as in Equation (1), with the addition of oral bioavailability limiting infant systemic exposure following maternal subcutaneous administration of the drug. S.C., subcutaneous.

However, rather than simply converting the weekly dose to an equivalent daily dose, the estimation of RID can be further extrapolated to estimate steady-state conditions over the full week by considering the plasma concentration-time profile of the mother. In the therapeutic dose range, semaglutide shows linear pharmacokinetic behavior. Overgaard et al. published population pharmacokinetic parameters of semaglutide [33]. According to this publication, the disposition of semaglutide after subcutaneous administration follows a 2-compartment model with first-order absorption from the injection site. These PK parameters were applied to simulate the plasma concentration-time course in the mother after repeated weekly dosing in Simulx 2024R1 (Lixoft SAS, Antony, France, a Simulations Plus company). Due to the long half-life of elimination of about one week, weekly dosing results in drug accumulation, i.e., the plasma concentrations at steady state are higher than the concentrations after the first dose. This is relevant for the estimation of RID, because the area under curve (AUC) in the mother’s plasma represents the input for the excretion into milk. The partial AUC in each 24-h time interval corresponds to the equivalent fraction of a single dose. The partial AUCs and associated daily dose equivalents vary over the weekly dosing interval according to the plasma concentration profile.

These fluctuations can be considered in the calculation of RID_F_ by introducing a corresponding factor, P_i,_, replacing the constant fraction of 1/7 in the denominator of Equation (2). This addition allows us to consider the slight increase in potential drug exposure from hours 24 to 72 after drug administration.

(3) $${RID}_{FP}(\%)=100\;\times \;\frac{infant\;daily\;oral\;dose\;per\;kg\;from\;milk\;\times \;F(oral)}{P_{i}*(maternal\;weekly\;subcutaneous\;dose\;per\;kg)\;\times \;F(S.C.)}$$

where P_i_ corresponds to the fractional dose equivalent on day i of the dosing interval (with weekly dosing, i = 1, 2…, 7). S.C., subcutaneous.

## 3. Results

### 3.1. Mass Spectrometry of Semaglutide

**Figure 1:**
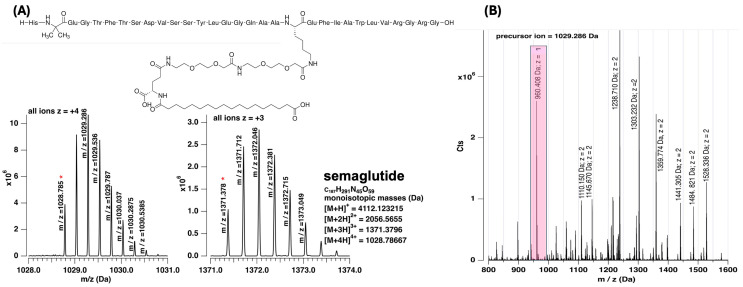
(**A**) MS spectrum of semaglutide illustrating isotopologues of [M + 4H^+^]^4+^ and [M + 3H^+^]^3+^ ions. (**B**) MS-MS spectrum of the z = +4, 1029.286 Da precursor ion. Highlighted ion at 960.408 Da was used for quantitation. The red asterisk (*) highlights selected precursor ions while the pink rectangle highlights the fragment used for quantitation.

Given the emergence of interest in GLP-1 agonists, it is not surprising that a variety of investigators have reported LC- MS/MS based assays for measurement of semaglutide in biological fluids [34,35,36,37,38,39]. Many of these studies have focused on exploring absorption, metabolism, or pharmacokinetics of the drug in plasma or urine although recent work studying the distribution of the drug in rodent brains has been attempted [40]. Similar to the current study, all efforts couple reversed phase liquid chromatography with electrospray ionization and tandem MS. The original method used the transition of 1029.1 → 136.0 Da, and this became the basis for subsequent work with a reported limit of quantification (LLOQ) of 3 ng/mL in plasma [35]. More recently, Lee et al. employed 1029.3 → 1302.9 Da for semaglutide mass analysis [40]. In the current work, we have chosen 1029.286 Da → 960.408 Da as the transition used for analysis. Figure 1 and the peak list found in Supplementary File S1 provide high resolution, high mass accuracy data of observed MS/MS peaks. We observed large fragment peaks at 1302.731 and 1303.232 Da similar to Lee et al.’s report of a fragment at 1302.9 Da while using a lower resolution mass spectrometer [40]. The current peak list reported within this manuscript is the highest resolution and most mass-accurate data set to date.

### 3.2. Chromatography and Figures of Merit

**Figure 2:**
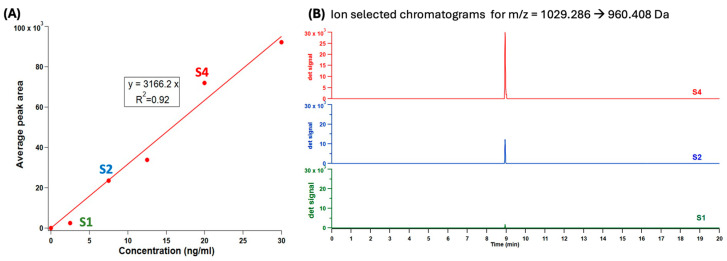
(**A**) Calibration curve of peak area vs. semaglutide concentration for 1029.286 → 960.408 Da transition. Semaglutide was spiked into human milk matrix. (**B**) Ion selected chromatograms of standard S1, S2, and S4 which demonstrate increased peak height and area with increasing semaglutide concentration.

We have developed a rapid LC-HR-MS method that allows the determination of the semaglutide in human milk in a 20-min run. Figure 2 reports both a calibration curve from 0–30 ng/mL semaglutide dissolved in human milk (Panel A), and three ion selected chromatograms (Panel B) corresponding to standards 1, 2, and 4. The ion selected chromatogram plots only the signal for the 1029.286 → 960.408 Da transition in time. One peak is noted in the chromatogram at approx. 8.9 min corresponding to elution of semaglutide. As observed in the figure, the average peak area for the selected transition scaled linearly with semaglutide concentration.

As indicated in Figure 2, a good linearity for the calibration curve ranging from 2.5–30 ng/mL of semaglutide was obtained, with a 3 s lower limit of detection (LLOD) of 1.7 ng/mL and a 10 s lower limit of quantification (LLOQ) of 5.7 ng/mL. It should be noted that these concentrations do not represent instrumental limits of detection, but rather the limits of analyte present within human milk matrix while having sample preparation protocol applied. For this particular study the LOD of 1.7 ng/mL was established through rigorous validation procedures, which included analyzing samples with known concentrations and assessing the signal to noise ratio. The 3 s standard for LOD was employed [41]. Thus, the limits stated essentially reflect our ability to detect analyte within human milk.

### 3.3. Analysis of Human Milk

**Figure 3:**
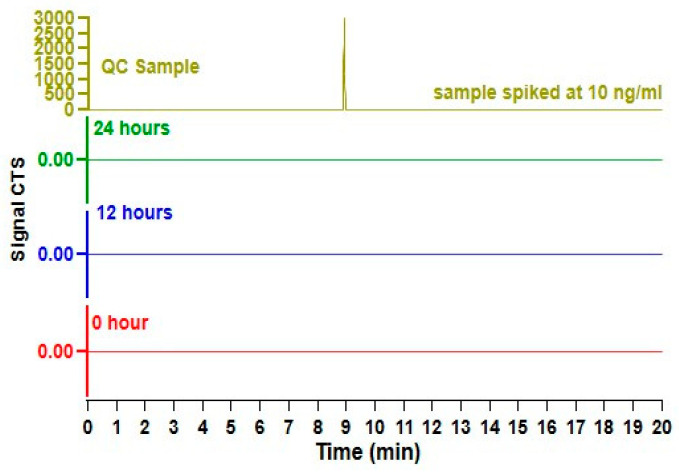
Chromatograms of samples collected at 0, 12, and 24 h post-semaglutide administration, compared with a quality control (QC) standard spiked at 10 ng/mL with semaglutide. No semaglutide was detected in the human milk.

The quantity of semaglutide present within milk at 0, 12, and 24 h after a self-administered subcutaneous injection is illustrated in Figure 3 and Table 1 below. We observed that at all time points, no semaglutide was detected for all human milk samples. This was confirmed by successful elution of quality control samples when spiked with known concentration of semaglutide assuring the reliability of the observed results.

### 3.4. Maternal and Infant Information

The self-reported maternal-infant health questionnaires collected at the time of milk sampling are summarized in Table 2. Half of the mothers were 1–2 years postpartum, while another 3/8 were more than 6 months postpartum. The participants represented a diverse racial profile. Although it is challenging to contextualize milk exposure this far postpartum, breast milk appeared to be a significant portion of the infant diet for most dyads, with minimal formula exposure. Despite the short exposure period for semaglutide (3 or more weeks during titration), mothers reported that all infants were meeting or surpassing expected milestones at the time of the survey, with infant growth being unremarkable. One mother reported that during a time she experienced gastrointestinal adverse effects from semaglutide, she was concerned her baby simultaneously experienced decreased appetite and diarrhea; however, she continued her usual breastfeeding regimen and ultimately concluded that there were no major effects on the infant’s weight changes or growth.

### 3.5. Pharmacokinetic Analysis

Semaglutide was not detected in any of the samples. As such, to estimate infant exposure to drug in milk, we will assume that the semaglutide concentration in milk at all time points was the lower limit of quantification, which will significantly overestimate the drug present in milk. The lower limit of quantification is the lowest concentration of analyte (semaglutide) that can be reliably measured using the method described above.

**Figure 4:**
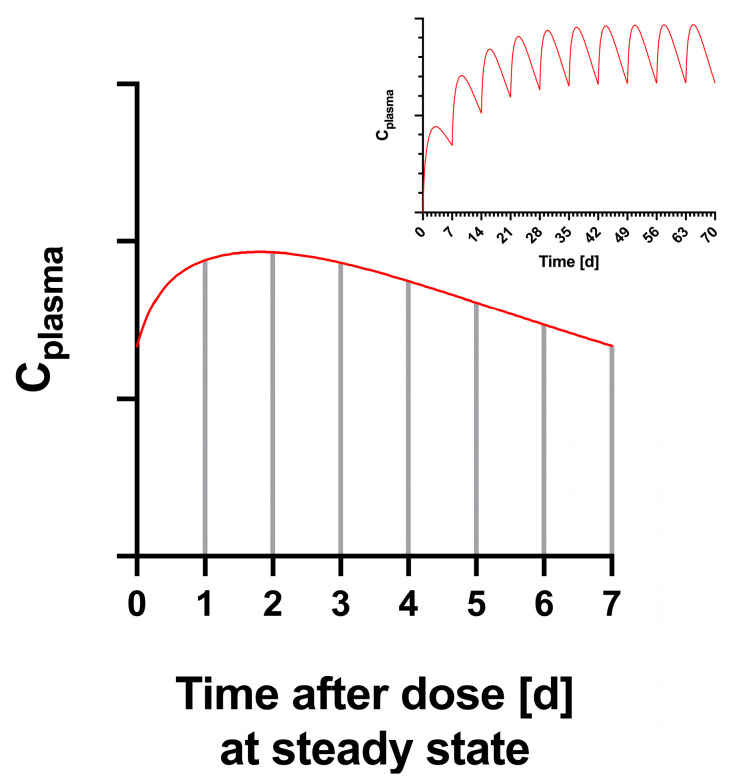
Relative plasma concentration time profile over a dosing interval at steady-state, and simulated profile over 10 weeks with weekly dosing (inset).

Using the reported LLOQ of 5.7 ng/mL and assuming an infant milk intake of 150 mL/kg/day, the maximum dose to the infant possible in this study without detecting semaglutide in milk was 855 ng/kg/day. Given the mean maternal weight of 93 kg delivering a mean 0.56 mg of semaglutide subcutaneously every week, the maximum RID_F_ possible given the undetectable levels in milk using the LLOQ is 1.12% (Table 3). Though we do not know how far actual milk concentrations of semaglutide fall below the LLOQ, no samples resulted in a measurable level. The simulated plasma concentrations (Figure 4) in the mother after weekly dosing indicated accumulation at steady-state by a factor of about 2.2.

Based on the plasma concentration-time profile at steady state, the fractional daily dose equivalents, as estimated from the partial AUC over the 24 h intervals (see Equation (3)) resulted in estimated RID_FP_ (%) values of less than 1.12, 1.26, 1.25, 1.18, 1.10, 1.01, 0.92 on day 1 through day 7, respectively, assuming the observed semaglutide concentration was at the LLOQ during the sampled time. The RID_FP_ (%) values reflect the slow, stable release profile of semaglutide over the dosing period.

## 4. Discussion

The maternal need for medications is a known risk factor for early breastfeeding cessation due to concerns of drug transfer into milk. While this concern is real, many healthcare providers and parents overestimate the theoretical risk of infant exposure to medications, while failing to fully consider the known opportunity costs associated with early breastfeeding cessation. The benefits of breastfeeding for the infant are well-known, though not always considered. For the mother, the benefits of lactation include a dose-intense reduction in some metabolic disorders [42]. These risk-benefit decisions have historically been handicapped by a lack of lactation research in physiology and pharmacology. As a result, though the Food and Drug Administration is approving these drugs without lactation data, they now issue post-marketing commitments for lactation data, including one with the approval of oral semaglutide tablets [43]. Unfortunately, for pregnancy, it takes about 11 years for this data to be included in product labeling [44]. This study was born out of an immediate clinical need for data in order to make evidence-informed decisions when treating breastfeeding women for type II diabetes and/or weight loss.

We found undetectable concentrations of semaglutide in breast milk at a LLOQ of 5.7 ng/mL and LLOD of 1.7 ng/mL. In an attempt to simulate worst-case scenarios of infant exposure, the LLOQ was used to estimate drug concentrations when estimating infant exposure. Using RID% to estimate infant exposure to a drug is an imperfect metric that has numerous drawbacks. Its interpretation is particularly vulnerable to changes in the maternal dose [26]. Further, the RID calculation was not designed for (1) injectable products with low oral bioavailability, and (2) it was not designed for products with extended dosing intervals. With no ideal way to estimate infant exposure via milk for a long-acting injectable drug with low oral bioavailability, the standard RID calculations were modified to include these parameters. The incredibly low oral bioavailability of the compound reduces risk, as seen by the extremely low 1.12% RID_F_ determined for semaglutide using the LLOQ. When adjusting the equation to account for a potential change in milk concentrations in the days after drug administration, the RID_FP_ only increased to a maximum of 1.26% on day 2. The authors believe that using the conventional practice of comparing RIDs against a 10% safety threshold, the findings support that the direct infant risk due to semaglutide in milk is likely negligible. While the low RID does not address all concerns surrounding maternal use of GLP-1 analogs, this finding answers the first pressing question: what risk of drug exposure does the breastfed infant have? In addition to milk concentrations of semaglutide, we also investigated maternally reported observations of the breastfeeding infant. The infants observed in this study ranged from 7–23 months of age. All mothers indicated that their infants were meeting or surpassing expected developmental milestones over a minimum of three weeks of exposure to the drug. However, one mother reported adverse gastrointestinal effects in herself and noted that her infant experienced transient diarrhea and decreased appetite during this period. This mother was temporarily concerned about a possible slowdown in her infant’s growth during the same timeframe, which she ultimately attributed to the increased caloric expenditure associated with the child’s new milestone of walking as the child continued tracking with their growth curve. While these reports are concerning, it is challenging to draw definitive conclusions from this observation, as such issues are also common, transient concerns during infant development. For context, a 2023 report on non-serious events in breastfed infants of mothers not taking medication found that 2.8% of healthy infants experienced poor feeding and 3.9% had diarrhea [45]. Ultimately, these findings highlight the need for further long-term investigation to accurately estimate the potential risk to the infant.

This study examined milk donated by participants but did not measure milk production or composition. Lactation typically occurs in a catabolic state as mothers mobilize stored fat from pregnancy. In overweight women, weight loss of approximately 0.5 kg per week does not affect the growth of their infants [46]. However, semaglutide may precipitate weight loss faster than is typically observed postpartum. This accelerated weight loss due to early satiety and restricted caloric consumption has the potential to reduce milk production and alter milk nutrient composition, which should be explored in future studies.

In some instances, the nutrient levels in breast milk remain stable despite changes in the mother’s nutritional status. However, ensuring a consistent supply of nutrients to the growing infant can deplete the mother’s reserves. Conversely, poor maternal nutrition and nutrient status can negatively affect the nutrient content of breast milk, potentially hindering the infant’s development [47]. To address these issues, breastfeeding mothers taking semaglutide should be monitored closely to ensure they meet daily nutrient recommendations based on the Dietary Reference Intakes (DRIs) established by the Health and Medicine Division of the National Academies of Sciences, Engineering and Medicine. Until these questions are answered, the maternal-infant dyad should be monitored closely if semaglutide is used. Dietary supplementation with a daily multivitamin for both the mother and the infant may be advisable to compensate for the potential insufficient maternal nutrient reserves and inadequate nutrient transfer through breast.

The presence of the GLP-1 hormone in human milk, contrasted with the observed lack of the GLP-1 analogue semaglutide in milk, raises important questions about the origin of GLP-1 in milk. If active GLP-1 were transported into human milk, we might expect semaglutide could be transported similarly. However, the absence of semaglutide suggests that GLP-1 may not be transported but rather produced within the mammary epithelium. Alternatively, the structural modifications of semaglutide could prevent its transport across the mammary epithelium, thus making it difficult to draw definitive conclusions about GLP-1 presence in milk. Given that GLP-1 is known to be produced by epithelial cells, it is plausible that mammary epithelial cells could be capable of the complex, multi-step production and posttranscriptional modification of proglucagon to GLP-1 [48].

The limitations of this study are important to consider when interpreting its findings. The small sample size of eight participants raises concerns about the generalizability of the results. Larger and more diverse samples would enhance the study’s external validity and strengthen the reliability of its measurements. While mothers were asked to report any infant adverse effects, long-term follow-up of the exposed infants would provide more comprehensive understanding of potential risks. This study examined milk donated by participants but did not measure milk production or composition. Accelerated weight loss has the potential to reduce milk production and alter milk nutrient composition and/or deplete maternal nutrients, which should be observed in future studies to determine the extent of this effect and related maternal and infant outcomes. Additionally, the study design only measured the early phases of dose titration in the donated milk. Examining the entire titration curve would be beneficial to determine the effects of higher drug dosages. The participants in this study were all in mature milk production or involution, as would be expected for postpartum patients with the intention of weight loss. The risk of elevated semaglutide transfer into milk and infant risk from drug exposure could be elevated during colostral phases; however, the authors believe semaglutide use is more likely later postpartum. Future studies should aim to detect semaglutide at lower quantifications in breast milk.

## 5. Conclusions

Our findings suggest that fears concerning infant exposure to maternal semaglutide via breast milk are likely overestimated. One mother observed potentially related transient diarrhea and decreased appetite in her infant, but the child’s growth curve was unaffected. It is important to note that extreme diets during lactation, which limit nutrient intake, can potentially reduce milk production and limit nutrient availability in the milk as well as deplete maternal nutrient stores. The negative impacts of weight gain during and after pregnancy on physical and mental health are well-documented. The nutrient intake of breastfeeding individuals considering semaglutide should be monitored closely, and a daily postnatal multivitamin may be considered for mother and child to ensure they meet the nutritional requirements supportive of lactation and development, respectively.

## Figures and Tables

**Table 1:** Summary of milk analysis results.

Participant	SubQ Dose (mg/Week)	Semaglutide (0 h)	Semaglutide (12 h)	Semaglutide (24 h)
1	0.5	n.d.	n.d.	n.d.
2	0.5	n.d.	n.d.	n.d.
3	0.25	n.d.	n.d.	n.d.
4	0.5	n.d.	n.d.	n.d.
5	0.25	n.d.	n.d.	n.d.
6	1	n.d.	n.d.	n.d.
7	0.5	n.d.	n.d.	n.d.
8 *	1	n.d.	n.d.	n.d.

* The participant reported using a semaglutide product from a compounding pharmacy rather than a product prepared by the manufacturer; n.d. means not detected at lower limit of detection (LLOD) of 1.7 ng/mL.

**Table 2:** Summary of self-reported maternal-infant health questionnaires.

Participant	Self-Reported Race	Weight (kg)	Dose Per Week	Maternal History	Maternal Meds	Infant History	Infant Age	Infant Diet	Infant Growth	Infant Milestones	Duration Infant Exposed to Milk with Semaglutide	Potentially Related Infant Adverse Events
1	white/caucasian	107	0.5	asthma, epilepsy, migraines, pcos, reynaud’s	prenatal, vitamin d, omega 3 fish oil,	dairy intolerance, poor growth until dairy removed from diet	12–23 months	breastmilk, water, solid foods (never formula)	average weight, height head circumference	right on track	4+ weeks	none
2	black & white	98	0.5	asthma, anxiety, depression GERD, gestational diabetes, PCOS	prenantal	none	7 to 11 months	breastmilk, water, solid foods (never formula)	average weight, height head circumference	right on track	4+ weeks	none
3	white	79	0.25	anemia, anxiety	none	dairy intolerance, frequent ear infection	7 to 11 months	breastmilk and some solid food (never forumla)	average weight and height, head circumference above average	surpassing expectations	4+ weeks	none
4	biracial (black/white)	112	0.5	infertility, PCOS	none	none	4 to 6 months	breastmilk and some rice cereal (never foruma, never solids)	average weight, height, and head circumference	right on track	9 weeks, interrupted	none
5	not reported	95	0.25	anxiety, asthma, depression, IBS	cetirizine, fluoxetine, buproprion, linaclotide, naltrexone	none	7 to 11 months	breastmilk, water, solid foods (never formula)	average weight, height, and head circumference	right on track	3+ weeks	none
6	white, non hispanic	97	1	migraines	none	none	12–23 months	breastmilk, water, juice, solid foods (never formula)	average weight, height, and head circumference	surpassing expectations	3+ weeks	possible diarrhea, possible decreased appetite (transient, resolved)
7	Black/African decent	83	0.5	anemia, anxiety, depression, high blood pressure, high cholesterol	Bupropion, desvenlafaxine, metformin	none	4 to 6 months	breastmilk every day, solid foods and formula on some days	average weight and height, head circumference well above average	right on track	4+ weeks	none
8 *	Mixed (Korean & White)	72	1	anxiety, depression, gestational diabetes, high cholesterol, PCOS	amphetamine salts, zolpidem, sertraline, alcaftadine eyedrops	none	12–23 months	breastmilk, formula, water, solid food every day	average weight, height, and head circumference	right on track	6+ weeks	none

* This mother reported that due to drug shortages, she was not able to keep her prescribed regimen when beginning her dose titration. GERD, Gastroesophageal Reflux Disease; PCOS, Polycystic Ovary Syndrome; IBS, Irritable Bowel Syndrome.

**Table 3:** Summary of Pharmacokinetic Parameters.

Parameter	Mean Value
Maternal Weight (kg)	93
Dose (mg/week)	0.56
C_avg_	n.d.
C_max_	n.d.
T_max_	unknown
LLOQ (ng/mL)	5.7
RID_F_ (%)	<1.12
RID_FP_ (%)	<1.26

n.d. means not detected at lower limit of quantification (LLOQ) of 5.7 ng/mL. Equation (2) was used to calculate RID_F_ assuming milk concentration was equal to the lower limit of quantification, which will overestimate the value. Equation (3) was used to calculate RID_FP_ using the same assumptions including *P_i_*; the maximum RID_FP_ is predicted to occur on Day 2.

## Data Availability

The raw data supporting the conclusions of this article will be made available by the authors on request.

## Supplementary Materials

The following supporting information can be downloaded at: https://www.mdpi.com/article/10.3390/nu16172886/s1, Supplementary File S1: Peak list for MS/MS analysis of semaglutide.
