# Supplementary material for: Subcutaneous Semaglutide during Breastfeeding: Infant Safety Regarding Drug Transfer into Human Milk
Source: Nutrients. 2024 Aug 28;16(17):2886. doi: 10.3390/nu16172886 (PMC11397063; doi:10.3390/nu16172886)
Supplement: Supplementary file 1 [file nutrients-16-02886-s001.zip › Feb 8 Milk Samples Results fpr semaglutide/221012B_0hour.pdf]

D:\breast milk project data\february8Palikasamples\221012B_0hour.... 2/9/2024 7:33:56 AM

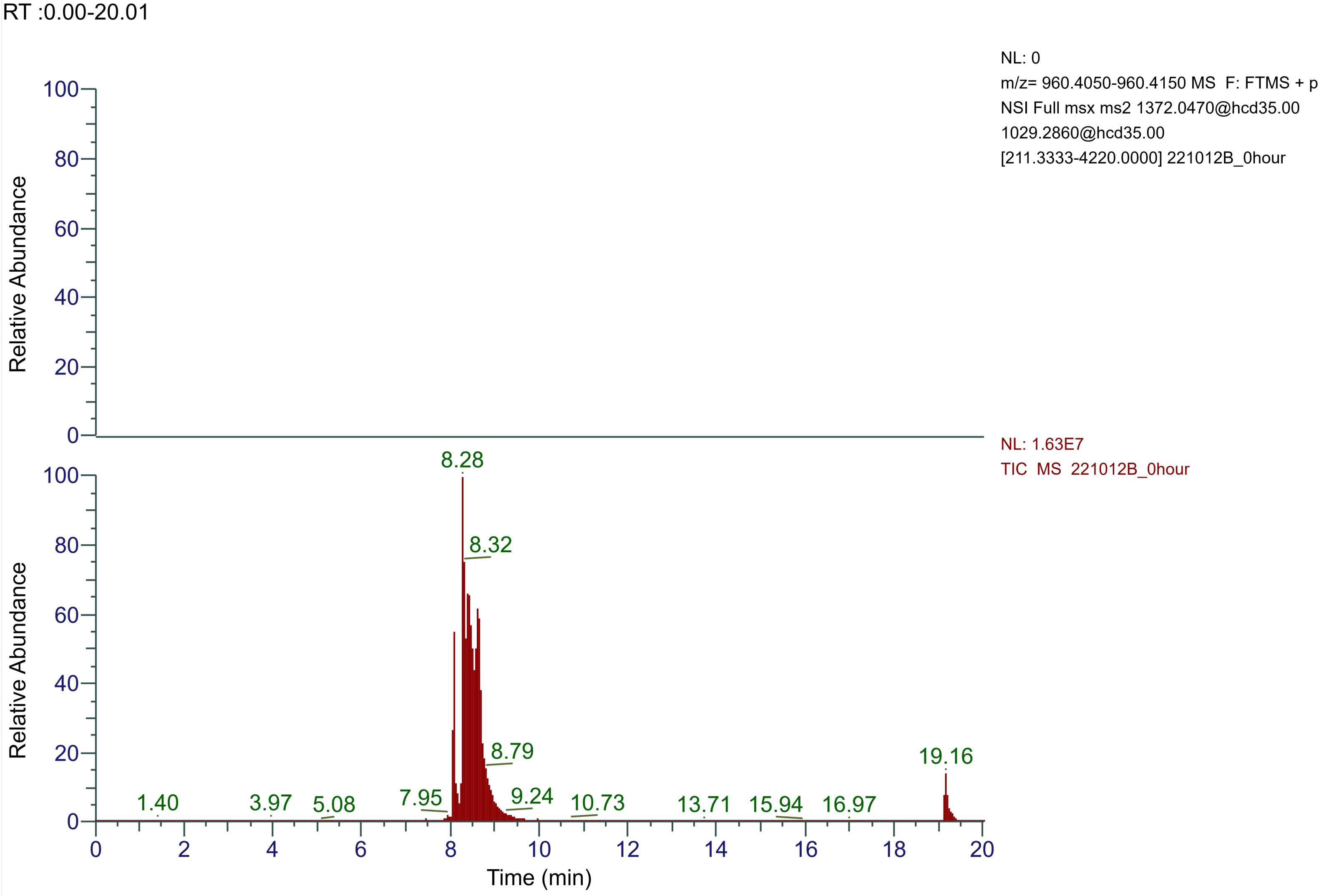

221012B_0hour #1446 RT: 8.30 AV: 1 NL: 4.70E5
T: FTMS + p NSI Full msx ms2 1372.0470@hcd35.00 1029.2860@hcd35.00 [211.3333-4220.0000]

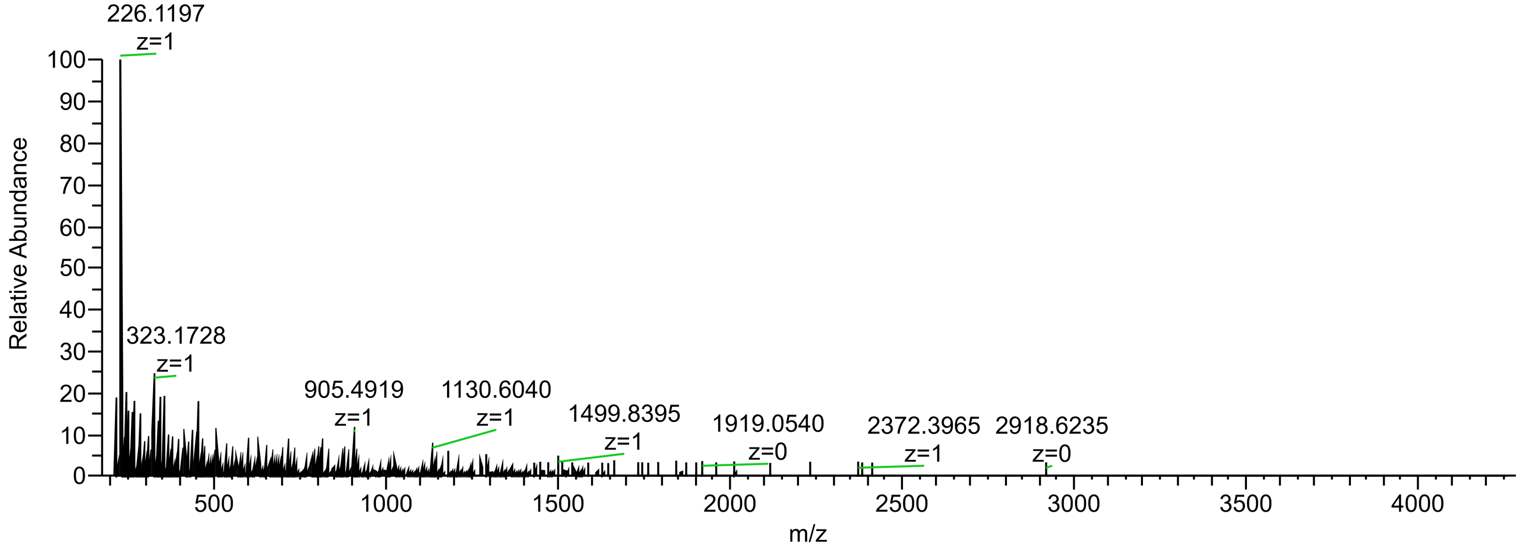

Display	File Name	Filter	Trace Type	Mass Def...	Ranges	Smoothi...	Chemical...	Mass Tol...	Plot Ope...	Trace Ty...	Range2	Comment
True	D:\breast milk project data \february 8Palikasa mples	FTMS + p NSI Full msx ms2 1372.047 0@hcd3 5.00 1029.286 0@hcd3	Mass Range	MDF Ranges	960.4100	Gaussian		5				
