# Supplementary material for: Subcutaneous Semaglutide during Breastfeeding: Infant Safety Regarding Drug Transfer into Human Milk
Source: Nutrients. 2024 Aug 28;16(17):2886. doi: 10.3390/nu16172886 (PMC11397063; doi:10.3390/nu16172886)
Supplement: Supplementary file 1 [file nutrients-16-02886-s001.zip › Feb 8 Milk Samples Results fpr semaglutide/230907B_24hour.pdf]

D:\breast milk project data\february8Palikasamples\230907B_24hou... 2/8/2024 4:43:26 PM

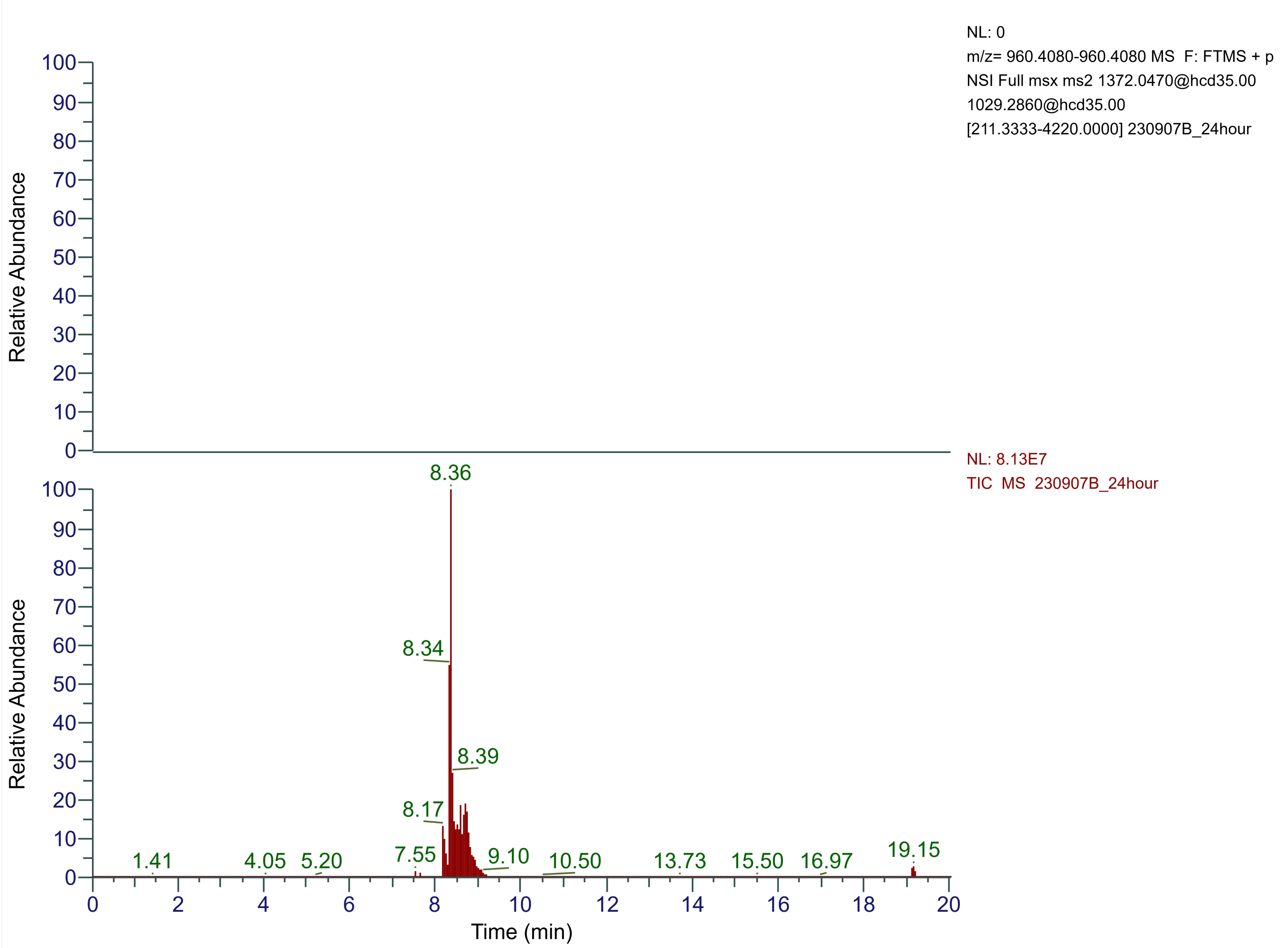

230907B_24hour #2631 RT: 15.11 AV: 1 NL: 1.89E3
T: FTMS + p NSI Full msx ms2 1372.0470@hcd35.00 1029.2860@hcd35.00 [211.3333-4220.0000]

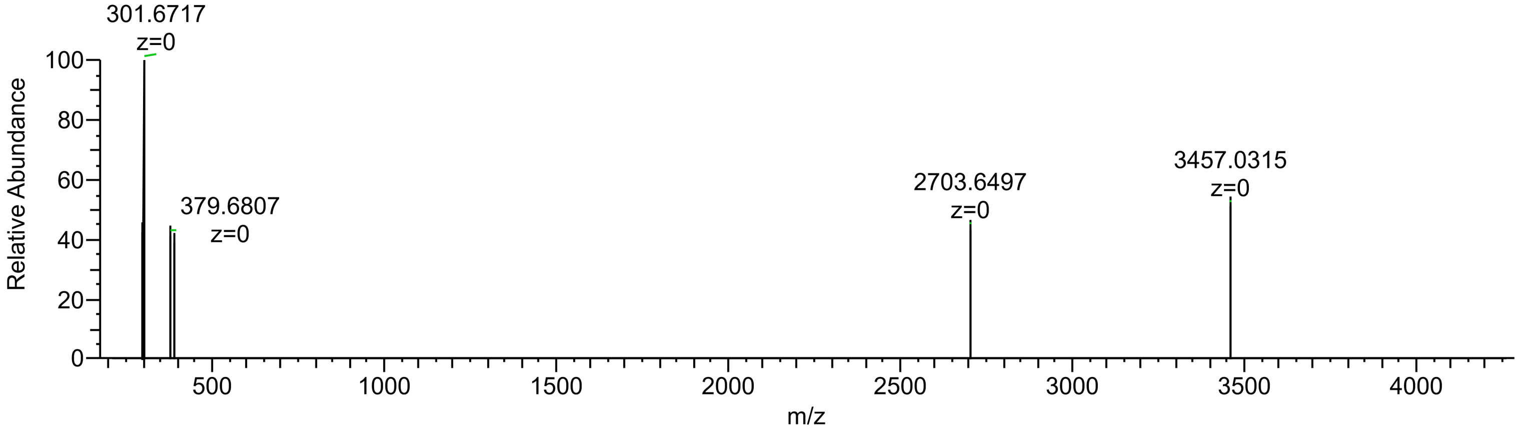

Display	File Name	Filter	Trace Type	Mass Def...	Ranges	Smoothi...	Chemical...	Mass Tol...	Plot Ope...	Trace Ty...	Range2	Comment
True	D:\breast milk project data\february8Palikasamples	FTMS + p NSI Full msx ms2 1372.0470@hcd35.00 1029.286	Mass Range	MDF Ranges	960.4080	Gaussian		5				
