# Supplementary material for: Subcutaneous Semaglutide during Breastfeeding: Infant Safety Regarding Drug Transfer into Human Milk
Source: Nutrients. 2024 Aug 28;16(17):2886. doi: 10.3390/nu16172886 (PMC11397063; doi:10.3390/nu16172886)
Supplement: Supplementary file 1 [file nutrients-16-02886-s001.zip › Feb 8 Milk Samples Results fpr semaglutide/230829A_12hour.pdf]

D:\breast milk project data\february8Palikasamples\230829A_12hou... 2/8/2024 1:03:35 PM

RT :0.00-12.17

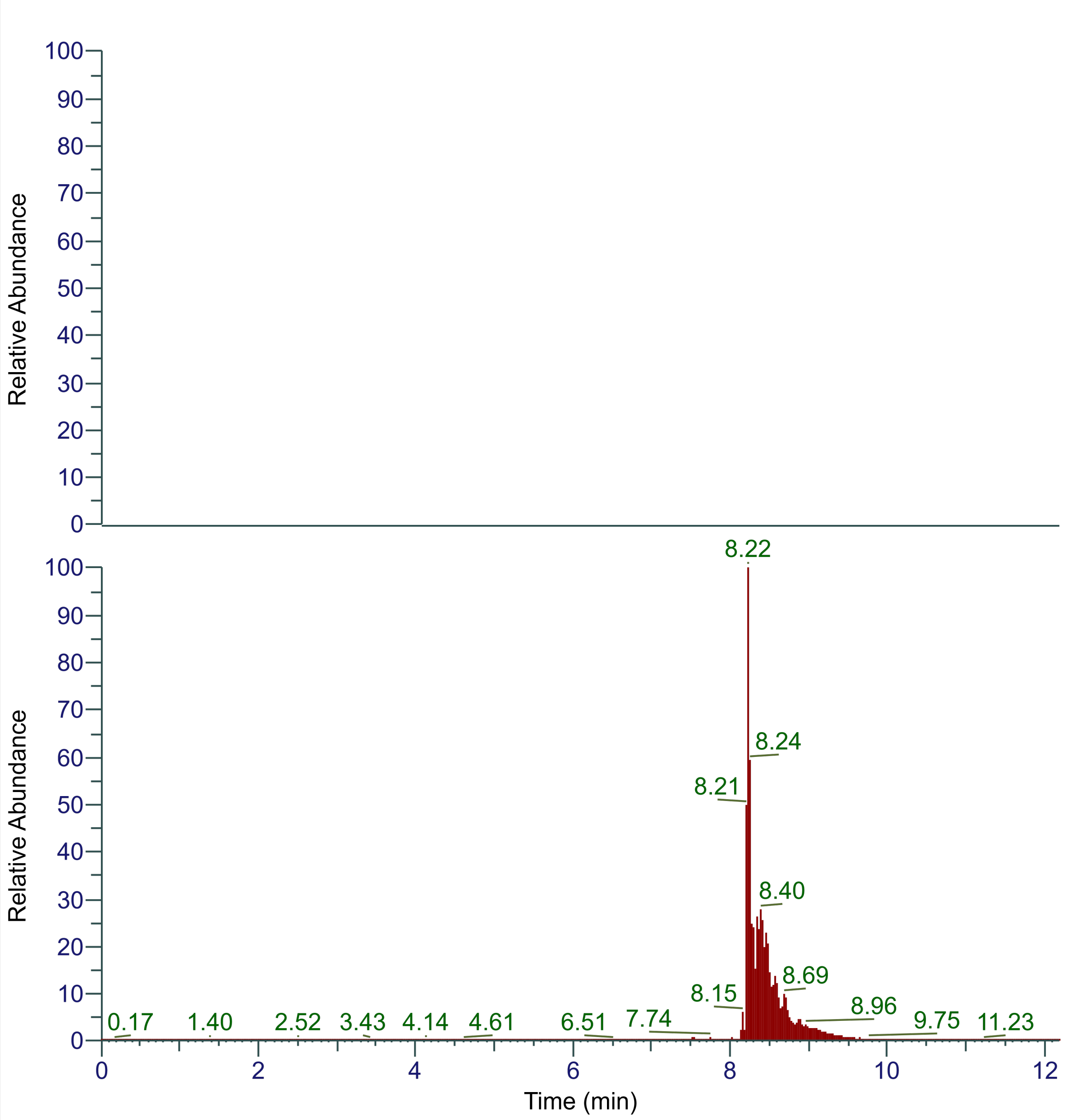

230829A_12hour #1432 RT: 8.22 AV: 1 NL: 4.16E5
T: FTMS + p NSI Full msx ms2 1372.0470@hcd35.00 1029.2860@hcd35.00 [211.3333-4220.0000]

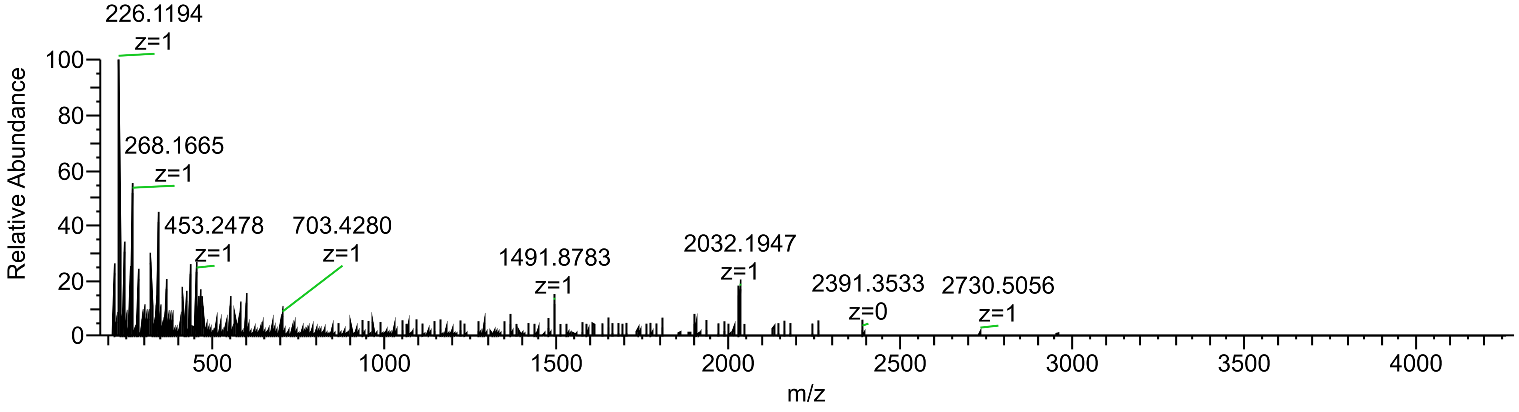

Display	File Name	Filter	Trace Type	Mass Def...	Ranges	Smoothi...	Chemical...	Mass Tol...	Plot Ope...	Trace Ty...	Range2	Comment
	D:\breast milk project data \february	FTMS + p NSI Full msx ms2 1372.047 0@hcd3	Mass	MDF		Gaussian						
