# Supplementary material for: Subcutaneous Semaglutide during Breastfeeding: Infant Safety Regarding Drug Transfer into Human Milk
Source: Nutrients. 2024 Aug 28;16(17):2886. doi: 10.3390/nu16172886 (PMC11397063; doi:10.3390/nu16172886)
Supplement: Supplementary file 1 [file nutrients-16-02886-s001.zip › Feb 8 Milk Samples Results fpr semaglutide/230829A_0hour.pdf]

D:\breast milk project data\february8Palikasamples\230829A_0hour.... 2/8/2024 1:01:54 PM

RT :0.00-20.01 SM: 7G

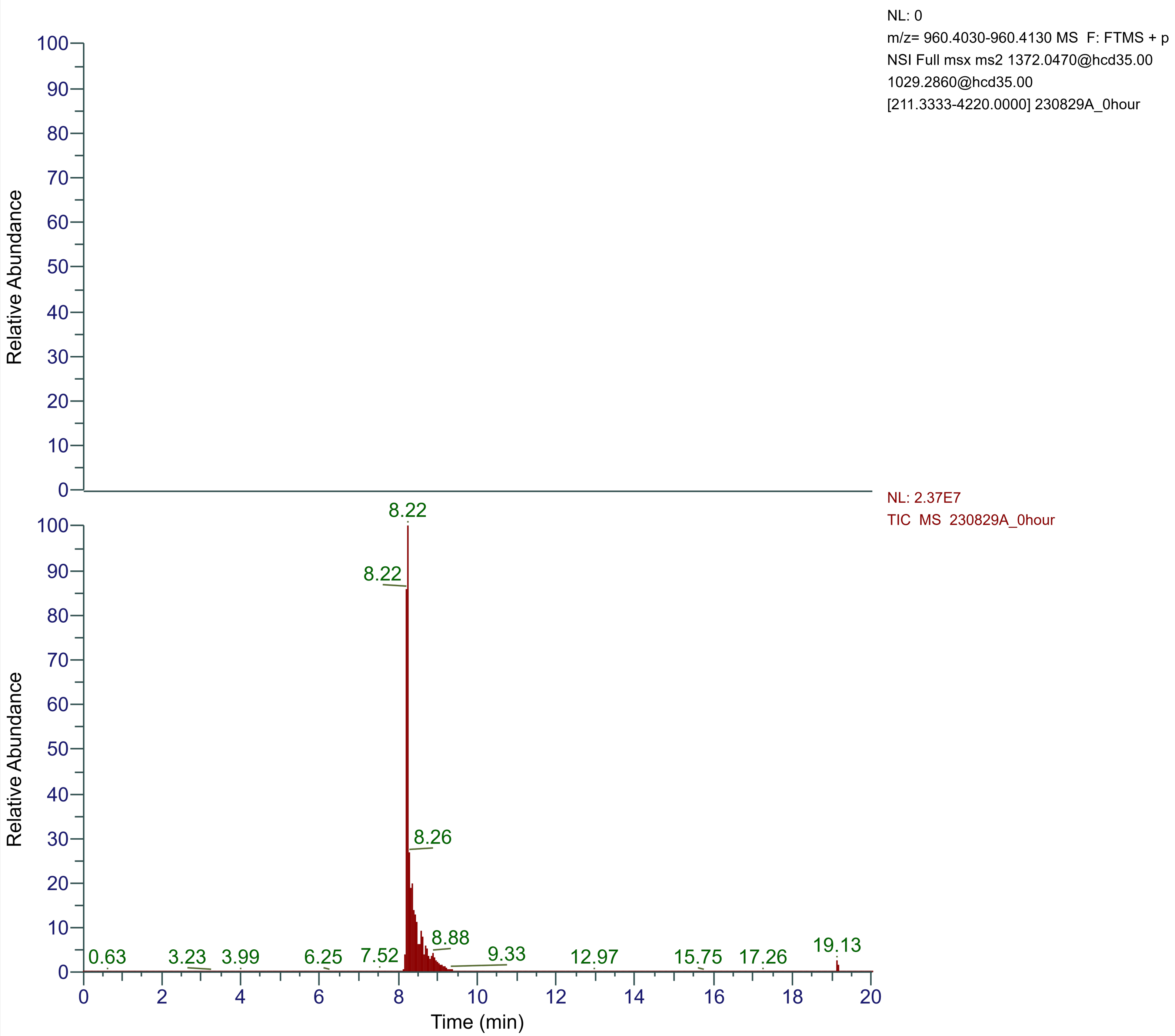

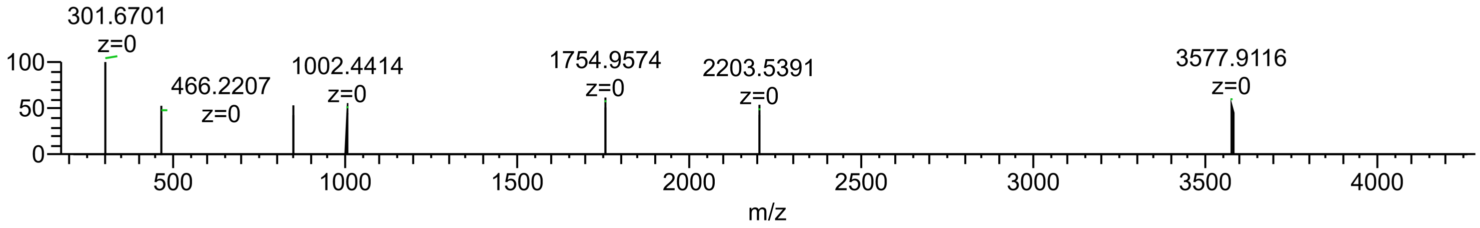

Display	File Name	Filter	Trace Type	Mass Def...	Ranges	Smoothi...	Chemical...	Mass Tol...	Plot Ope...	Trace Ty...	Range2	Comment
True	D:\breast milk project data \february 8Palikas[illegible]	FTMS + p NSI Full msx ms2 1372.047 0@hcd3 5.00	Mass Range[illegible]	MDF Ranges[illegible]	960.4080	Gaussian 7		5				
