# Supplementary material for: Subcutaneous Semaglutide during Breastfeeding: Infant Safety Regarding Drug Transfer into Human Milk
Source: Nutrients. 2024 Aug 28;16(17):2886. doi: 10.3390/nu16172886 (PMC11397063; doi:10.3390/nu16172886)
Supplement: Supplementary file 1 [file nutrients-16-02886-s001.zip › Feb 8 Milk Samples Results fpr semaglutide/s5_late.pdf]

D:\breast milk project data\february8Palikasamples\s5_2024020900... 2/9/2024 7:55:43 AM

RT :7.36-9.81 SM: 7G

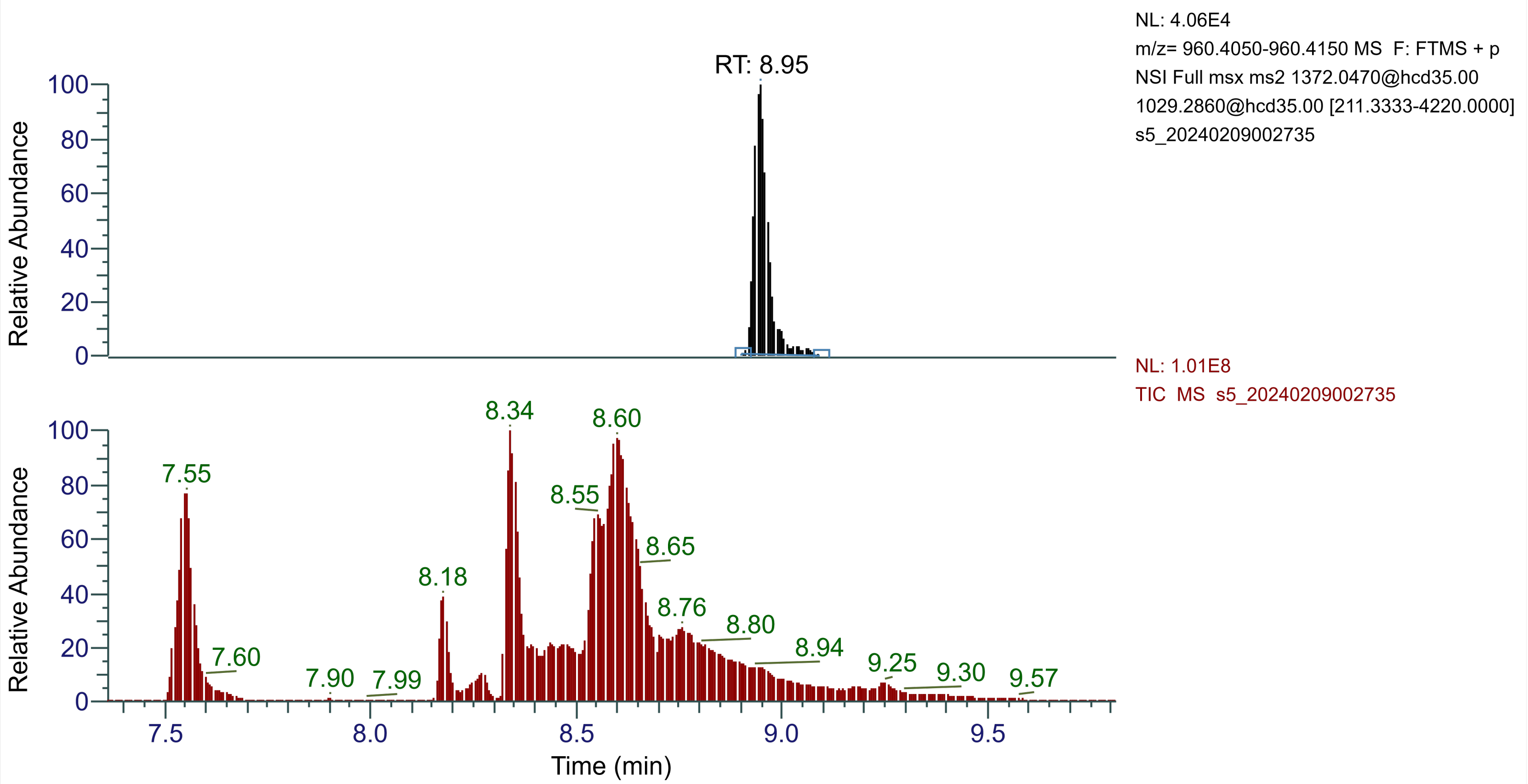


s5_20240209002735 #1553-1567 RT: 8.92-9 AV: 15 NL: 3.53E4

T: FTMS + p NSI Full msx ms2 1372.0470@hcd35.00 1029.2860@hcd35.00 [211.3333-4220.0000]

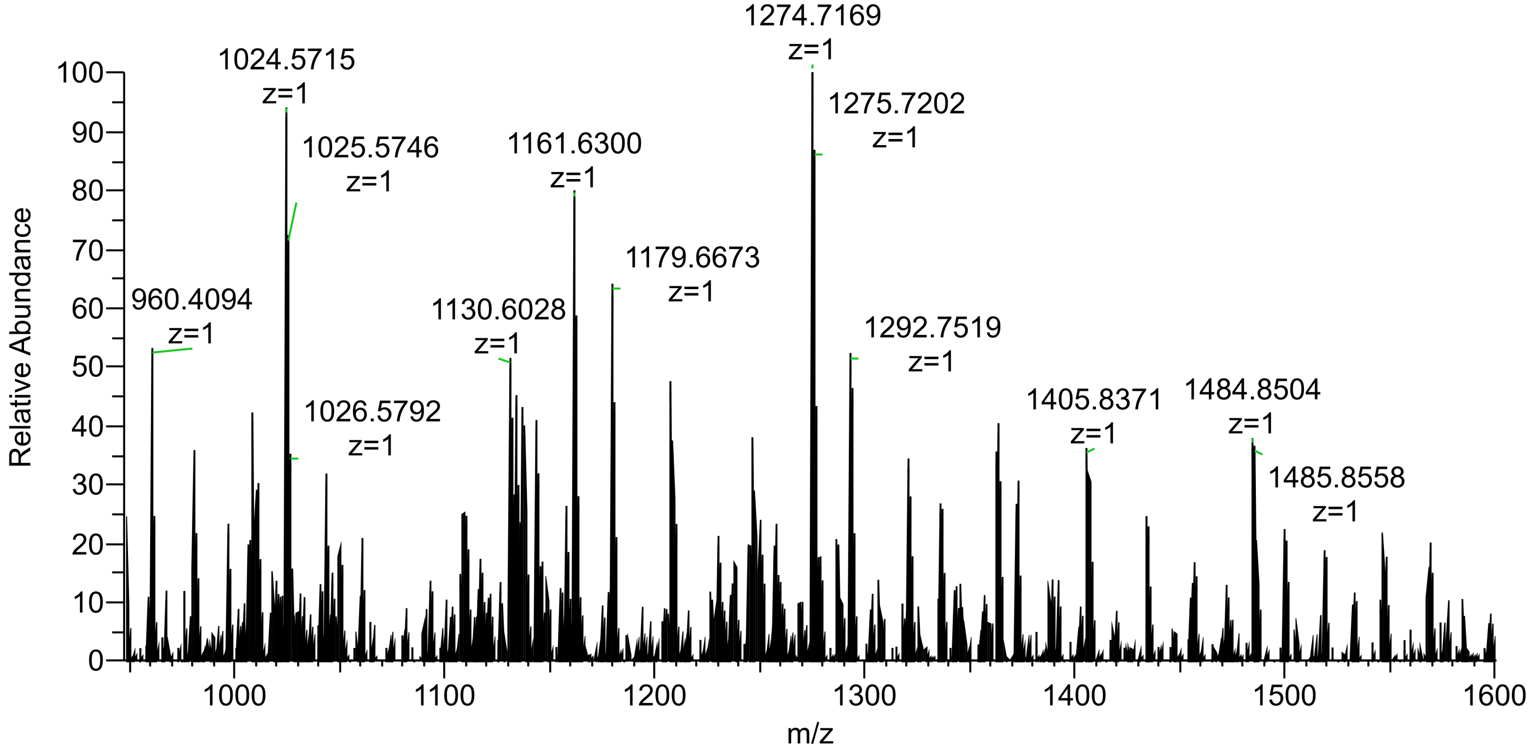


| Display | File Name | Filter | Trace Type | Mass Def... | Ranges | Smoothi... | Chemical... | Mass Tol... | Plot Ope... | Trace Ty... | Range2 | Comment |
|---|---|---|---|---|---|---|---|---|---|---|---|---|
| True | D:\breast milk project data \february 8Palikasa mples | FTMS + p NSI Full msx ms2 1372.047 0@hcd3 5.00 1029.286 | Mass Range | MDF Ranges | 960.4100 | Gaussian 7 | | 5 | | | | |
