# Supplementary material for: Subcutaneous Semaglutide during Breastfeeding: Infant Safety Regarding Drug Transfer into Human Milk
Source: Nutrients. 2024 Aug 28;16(17):2886. doi: 10.3390/nu16172886 (PMC11397063; doi:10.3390/nu16172886)
Supplement: Supplementary file 1 [file nutrients-16-02886-s001.zip › Feb 8 Milk Samples Results fpr semaglutide/231117A_0hour.pdf]

D:\breast milk project data\february8Palikasamples\231117A_0hour.... 2/8/2024 6:19:17 PM

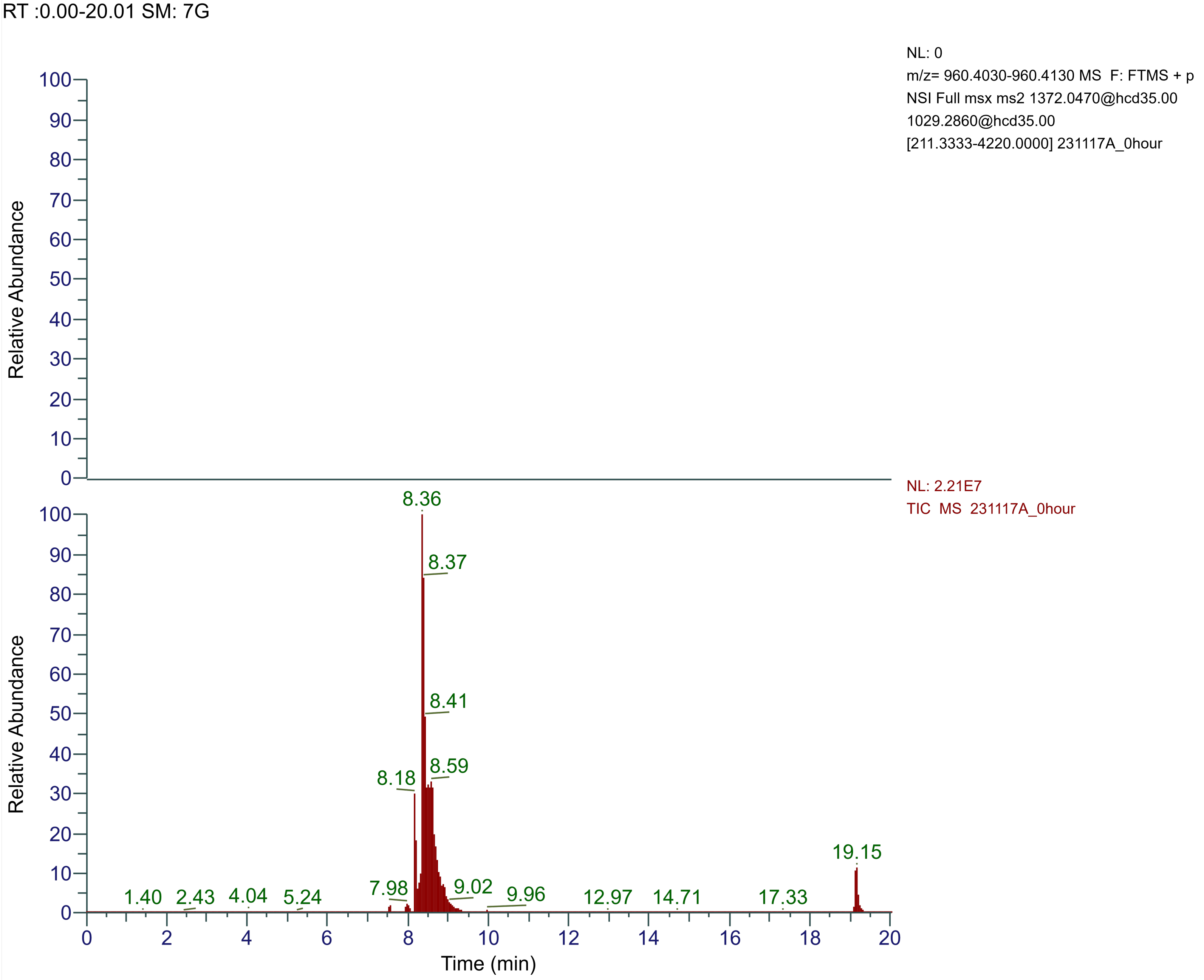

231117A_0hour #1 RT: 0.01 AV: 1 NL: 1.37E3
T: FTMS + p NSI Full msx ms2 1372.0470@hcd35.00 1029.2860@hcd35.00 [211.3333-4220.0000]

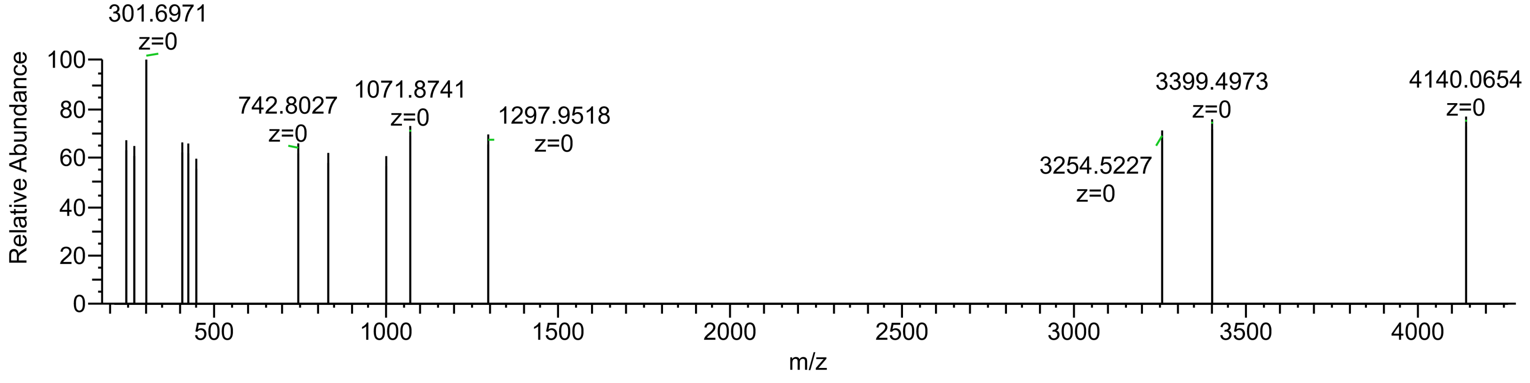

Display	File Name	Filter	Trace Type	Mass Def...	Ranges	Smoothi...	Chemical...	Mass Tol...	Plot Ope...	Trace Ty...	Range2	Comment
True	D:\breast milk project data \february [illegible]	FTMS + p NSI Full msx ms2 1372.047 0@hcd3 5.00 [illegible]	Mass [illegible]	MDF [illegible]	960.4080	Gaussian [illegible]		5				
