# Supplementary material for: Subcutaneous Semaglutide during Breastfeeding: Infant Safety Regarding Drug Transfer into Human Milk
Source: Nutrients. 2024 Aug 28;16(17):2886. doi: 10.3390/nu16172886 (PMC11397063; doi:10.3390/nu16172886)
Supplement: Supplementary file 1 [file nutrients-16-02886-s001.zip › Feb 8 Milk Samples Results fpr semaglutide/QC10lateR.pdf]

D:\breast milk project data\february8Palikasamples\Palika_QC10_20... 2/9/2024 7:45:40 AM

RT :6.49-13.50 SM: 7G

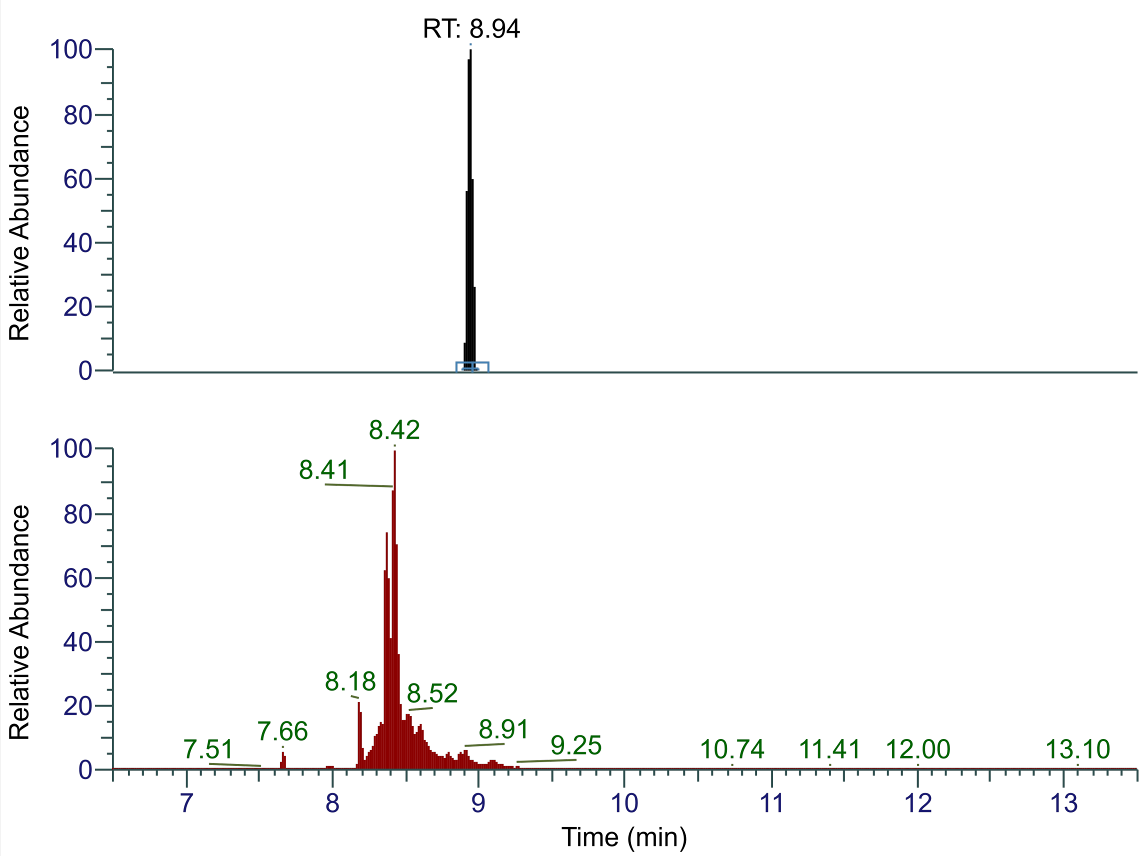

NL: 2.99E3
m/z= 960.4050-960.4150 MS F: FTMS + p NSI Full msx ms2 1372.0470@hcd35.00 1029.2860@hcd35.00 [211.3333-4220.0000] Palika_QC10_20240208223521

NL: 3.14E7
TIC MS Palika_QC10_20240208223521

Palika_QC10_20240208223521 #1552-1562 RT: 8.91-8.97 AV: 11 NL: 3.85E3
T: FTMS + p NSI Full msx ms2 1372.0470@hcd35.00 1029.2860@hcd35.00 [211.3333-4220.0000]

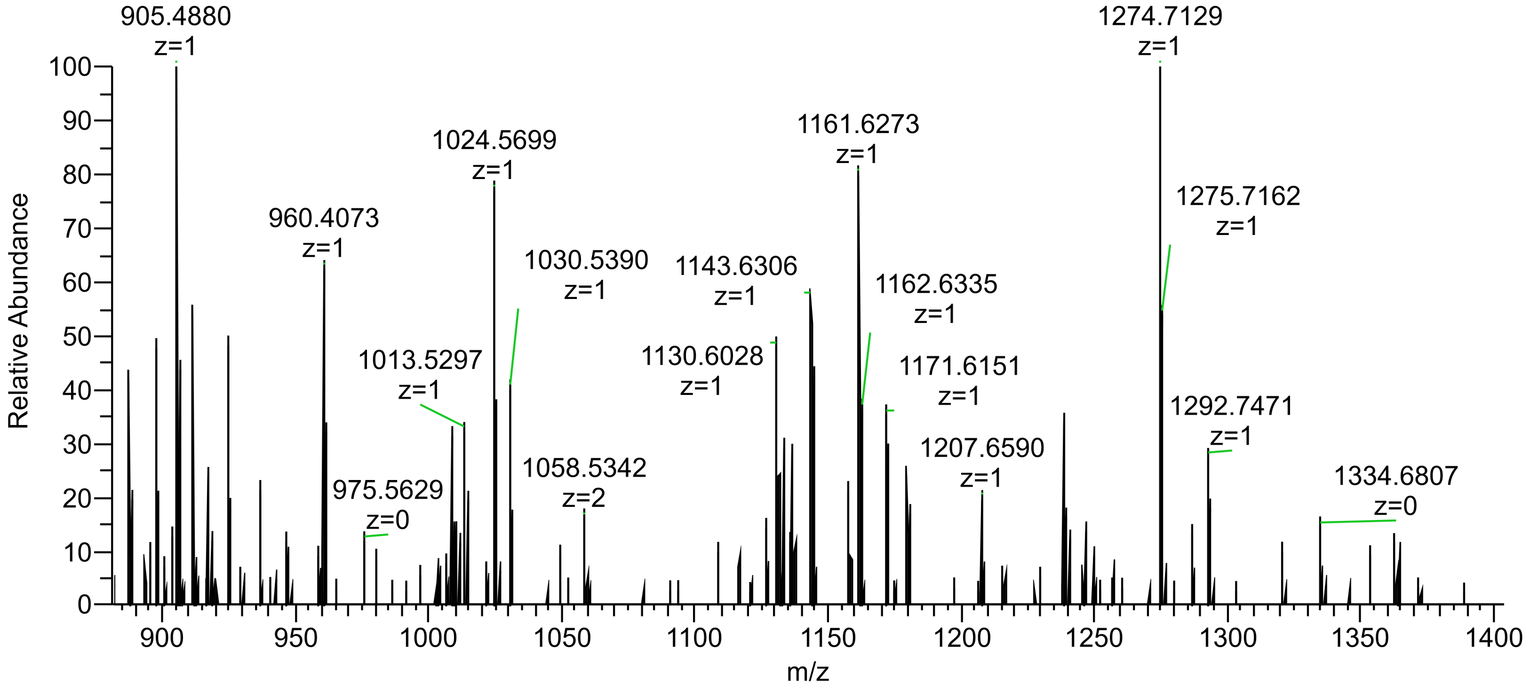

Display	File Name	Filter	Trace Type	Mass Def...	Ranges	Smoothi...	Chemical...	Mass Tol...	Plot Ope...	Trace Ty...	Range2	Comment
True	D:\breast milk project data \february 8Palikasa mples [illegible]	FTMS + p NSI Full msx ms2 1372.047 0@hcd3 5.00 1029.286 [illegible]	Mass Range	MDF Ranges	960.4100	Gaussian 7		5				
