# Supplementary figures and images for: Subcutaneous Semaglutide during Breastfeeding: Infant Safety Regarding Drug Transfer into Human Milk
Source: Nutrients. 2024 Aug 28;16(17):2886. doi: 10.3390/nu16172886 (PMC11397063; doi:10.3390/nu16172886)

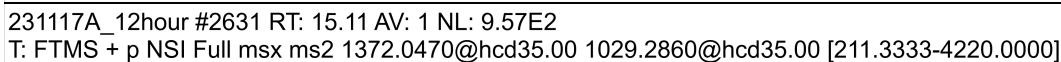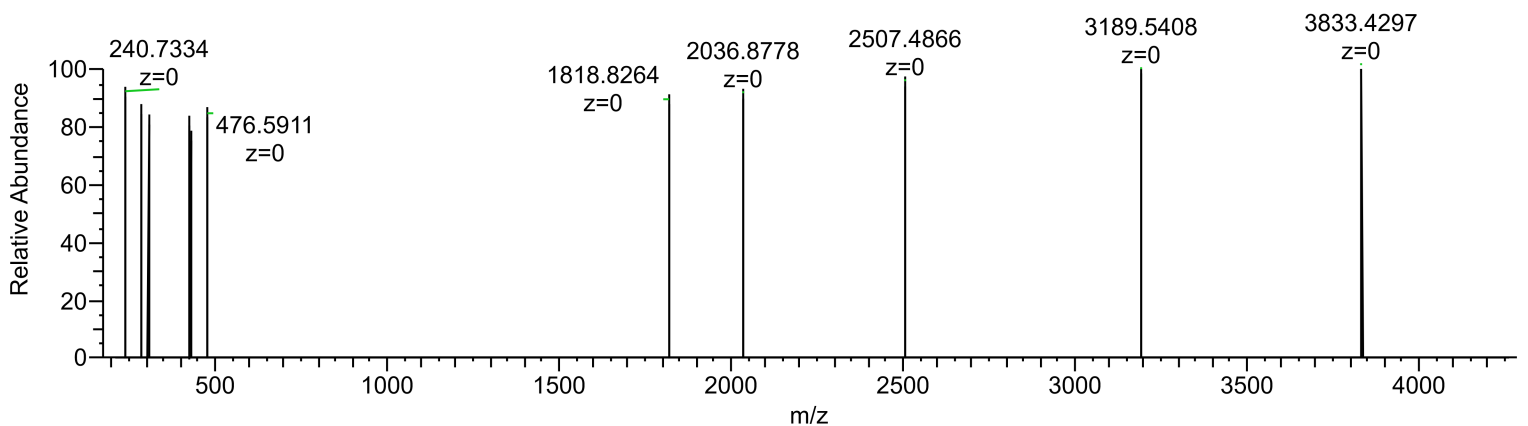[illegible]

Supplement: Supplementary file 1 [file nutrients-16-02886-s001.zip › Feb 8 Milk Samples Results fpr semaglutide/231117A_12hour.pdf]
